# Internet gaming disorder and aggression: A meta-analysis of teenagers and young adults

**DOI:** 10.3389/fpubh.2023.1111889

**Published:** 2023-04-06

**Authors:** Shunyu Li, Zhili Wu, Yuxuan Zhang, Mengmeng Xu, Xiaotong Wang, Xiaonan Ma

**Affiliations:** ^1^Center for Higher Education Development Research in Xinjiang, Xinjiang Normal University, Ürümqi, Xinjiang, China; ^2^Department of Social and Behavioural Sciences, City University of Hong Kong, Kowloon, Hong Kong SAR, China; ^3^School of Education, Liaoning Normal University, Dalian, Liaoning, China

**Keywords:** internet gaming disorder, aggression, meta-analysis, teenagers and young adults, online game addiction

## Abstract

**Background and aims:**

Internet gaming disorder (IGD) and aggression (AG) are widespread phenomena around the world. Numerous studies have explored the relationship between the two but findings from such studies are inconsistent. The meta-analysis aimed to evaluate the relationship between IGD and AG as well as identify the variables moderating the relationship.

**Method:**

Studies investigating the relationship between IGD and AG were searched using selected terms to identify studies published from 1999 to 2022 on CNKI, Wanfang Data, Chongqing VIP Information Co., Ltd. (VIP), Baidu scholar, ProQuest dissertations, Taylor & Francis, Springer, Web of Science, Google Scholar, Elsevier Science (Science Direct), EBSCO, and PsycINFO. The identified studies were pooled and analyzed.

**Results:**

A total of 30 samples comprising 20,790 subjects were identified. Results showed that there was a moderate relationship between IGD and AG (*r* = 0.300, 95%CI [0.246, 0.353]). Moderator analysis revealed that the relationship between IGD and AG was moderated by the region, age, and survey year.

**Conclusion:**

This meta-analysis indicated that people with a higher level of IGD might show more aggression, and people with more aggression might have a higher level of IGD. The correlation coefficient between IGD and AG was significantly higher in Asia than in Europe, higher in primary school than in middle school and university, and higher by increasing year. Overall, our findings provide a basis for developing prevention and intervention strategies against IGD and AG.

**Systematic review registration:**

https://www.crd.york.ac.uk/prospero/display_record.php?ID=CRD42022375267, 42022375267.

## 1. Introduction

According to a survey by the United Nations Educational, Scientific and Cultural Organization (UNESCO), 32% of students (1) and about 246 million children experience physical violence and bullying at school every year (2). Globally, youth violence and bullying are major challenges affecting the public health and education sectors (2, 3). Bullying and violence are forms of aggression (AG) (4). The prevalence of AG has been on the increase annually (5, 6). AG is detrimental to an individual’s physical and mental health as well as career development (7, 8). Therefore, researchers should explore avenues for controlling and reducing the rates of AG.

Previous studies mainly evaluated the causes of AG from personal and environmental perspectives (9–13). Internet games belong to virtual environments. Adolescents are prone to addiction to the virtual online world and their rate of addiction has been increasing yearly (14, 15). Therefore, Internet Gaming Disorder (IGD) is considered an influencing factor for development of aggressive behaviors among adolescents (16, 17). Some empirical studies have shown that IGD was highly associated with AG (18–20). This is possibly because aggressive individuals have poor interpersonal relationships and low self-efficacy in real life, therefore they seek their self-worth and obtain self-efficacy through virtual Internet games (21). Players may acquire the feeling by engaging in games, which makes their behaviors repetitive, leading to IGD (22). On the other hand, IGD may have various negative impacts including aggression, hostility, and antagonistic behaviors (23), which may be explained by the General Aggression Model (24). Aggressive characteristics are part of a person’s personality, and are correlated with immediate aggressive behaviors and long-term aggressive personalities (25). Among the Internet games, students tend to prefer violent games (26). Negative scenarios in Internet games can trigger attacks and hostile behaviors among individuals in real life. In contrast, some studies reported weak relationships (27, 28) or even a negative correlation between IGD and AG (29). This can be explained by the catharsis theory of play which states that individuals use Internet games to reduce stress or satisfy controlling needs that have not been met in real life. Gamers use online games as a means to reduce stress, and some stressed, unhappy, or mentally ill people are more inclined to use Internet games to release stress (30). People who play more games are better capable of handling stressful tasks in laboratory settings (31). Young moderate gamers tend to have better mental health outcomes than non-gamers or excessive gamers (32). As a result, the negative emotions and aggressive behaviors may be moderately relieved.

The nature of the relationship between IGD and AG has not been conclusively determined, which may be due to the small number of participants in single surveys. Therefore, we performed this meta-analysis to integrate previous empirical studies of IGD and AG to provide stronger scientific conclusions about the relationship between IGD and AG.

Inconsistencies in IGD and AG relationships among studies may also be attributed to differences in measurement tools used for IGD and AG as well as differences in subjects’ demographic characteristics (33, 34). We hypothesized that the relationship between IGD and AG is affected by one or more variables. Specifically, this relationship may be influenced by: (i) The choice of IGD and AG measures; (ii) Demographic profiles of participants. We explored the moderating role of the four demographic variables: year, gender, region, and age.

### 1.1. Measures of IGD and AG

In 2013, the American Psychiatric Association (APA) first included IGD in the Diagnostic and Statistical Manual of Mental Disorders Fifth Edition (DSM-5). DSM-5 considers IGD to be an excessive and prolonged mode of Internet gaming in which individuals with IGD experience exhibit multiple cognitive and behavioral symptoms, such as gradual loss of control over gaming, tolerance, and withdrawal symptoms. It contains 9 diagnostic criteria: Addiction to Internet games; Withdrawal symptoms; Tolerance; Failed attempts to control Internet games; Loss of interest in other activities; Ignoring existing psychosocial problems and continuing to overuse online games; Deception for the sake of the game; Escape from destructive emotions; Endangering or losing an important relationship or opportunity for work or education due to online games. A person who meets five or more criteria in the past 12 months is considered to be suffering from IGD (35). Based on the nine diagnoses of IGD in DSM-5, various assessment tools for IGD have been developed. For example, Pontes and Griffiths developed the nine-item Internet Gaming Disorder Scale—Short-Form (IGDS9-SF) (36). Lemmens and colleagues developed 4 questionnaires including two long scales (27 items) and two short scales (9 items) that have good reliability and validity (37). These scales can be divided into two types: polytomous and dichotomous. Later, Lemmens compiled another Game Addiction Scale (GAS), which includes a complete version consisting of 21 factors and a short version consisting of 7 factors. Both scales showed good reliability and validity (38). One of the most widely used IGD scales in the Chinese mainland is Cui’s Internet addiction diagnostic scale (IADS), which was developed based on Young’ Diagnostic Questionnaire and revised by the Angoff method (39, 40).

Aggression refers to actions that are intended at physically or psychologically harming others (41). When studying the relationship between IGD and AG, the early and frequently used AG measurement tool is the Buss-Durkee Hostility Inventory (BDHI). The BDHI which includes seven assault dimensions (indirect hostility, irritability, negativism, resentment, suspicion, verbal hostility and guilt), is used to assess the intensity and performance of hostility as well as AG (42). Since this scale did not perform factor analysis on each item, Buss and Perry compiled the BPAQ based on BDHI to improve the performance of the AG assessment tool (43). It consists of 29 questions with four dimensions: physical aggression, verbal aggression, anger and hostility. The BPAQ has been verified and is used worldwide. Other commonly used AG scales include the Reactive-Proactive Aggression Questionnaire (44, 45), the Normative Beliefs About Aggression Scale (46), Barratt Impulsiveness Scale (47) and State–Trait Anger Expression Inventory (48).

In summary, different measurement tools have different theoretical, dimension constructions and number of questions. These differences may have an impact on the relationship between IGD and AG to a certain extent. Therefore, we analyzed the moderating effects of measurement tools on IGD and AG.

### 1.2. Demographic variables as moderators

Demographic variables include the region, age, year and gender of the subject. Differences across regions may cause significant differences in the relationship between IGD and AG. Studies in Asia and Europe have reported contrasting findings on correlations between IGD and AG (18, 49, 50). For instance, some studies in Asia reported moderate positive correlations between IGD and AG (51, 52). However, in Europe, a low degree of positive correlation between IGD and AG was reported (27). Therefore, we hypothesized that the relationship between IGD and AG varies significantly across regions.

Differences in age may lead to significantly different relationships between IGD and AG. Among college and middle school students, the IGD and AG correlate to varying degrees (19, 52). Some studies reported a low positive correlation between IGD and AG among middle school and college students (27, 28). Other studies found a moderate positive correlation between IGD and AG in middle school and college students (18, 53). Therefore, we explored whether there are significant age-associated differences with in terms of the relationship between IGD and AG.

Gender differences can also cause significant differences in the relationship between IGD and AG. Different correlations between male and female students with IGD and AG have been reported (51, 53). Some studies reported low positive correlations between men’s IGD and AG (28), whereas others proved that women’s IGD and AG are moderately positively correlated (54). Therefore, we investigated whether there are significant gender-associated differences in the relationship between IGD and AG.

Finally, the year may be a moderating variable affecting the relationship between IGD and AG. Some studies have shown that the correlation between IGD and AG increases annually (28, 49, 55) whereas other studies reported that the relationship between IGD and AG decreases each year (20, 50, 56). Therefore, we investigated the differences in IGD and AG between students in different years.

Therefore, we conducted a meta-analysis on studies investigating the relationship between IGD and AG to: (i) Determine the effect size and direction of the relationship between IGD and AG and (ii) Determine how various factors (measurement tools, region, age, gender, year) affect the relationship between IGD and AG?

## 2. Methods

This meta-analysis was performed in accordance with the Preferred Reporting Items for Systematic reviews and Meta-Analyses (PRISMA) statement. To increase transparency and prevent unintended duplication of efforts, the protocol used in this meta-analysis was preregistered at the International Prospective Register for Systematic Reviews (PROSPERO) (CRD:42022375267).

### 2.1. Literature search

We searched the CNKI, Wanfang Data, Chongqing VIP Information Co., Ltd. (VIP), Baidu scholar, ProQuest dissertations, Taylor & Francis, Springer, Web of Science, Google Scholar, Elsevier Science (Science Direct), EBSCO and PsycINFO databases to retrieve relevant studies investigating the relationship between AG and IGD published from January 1999 to November 2022. The key search terms for IGD were: online game addiction, Internet game disorder, digital game addiction, problematic Internet game use, Internet game dependence, video game addiction, excessive Internet game use and computer game addiction. The main search terms for AG were: aggression, impulsiveness, anger, conflict, attack, hostility, violence, aggressiveness, aggressive action, aggressive behavior, behavior disorder, behavior problems, conduct disorder, anti-social behavior and oppositional defiant disorder.

**Figure 1:**
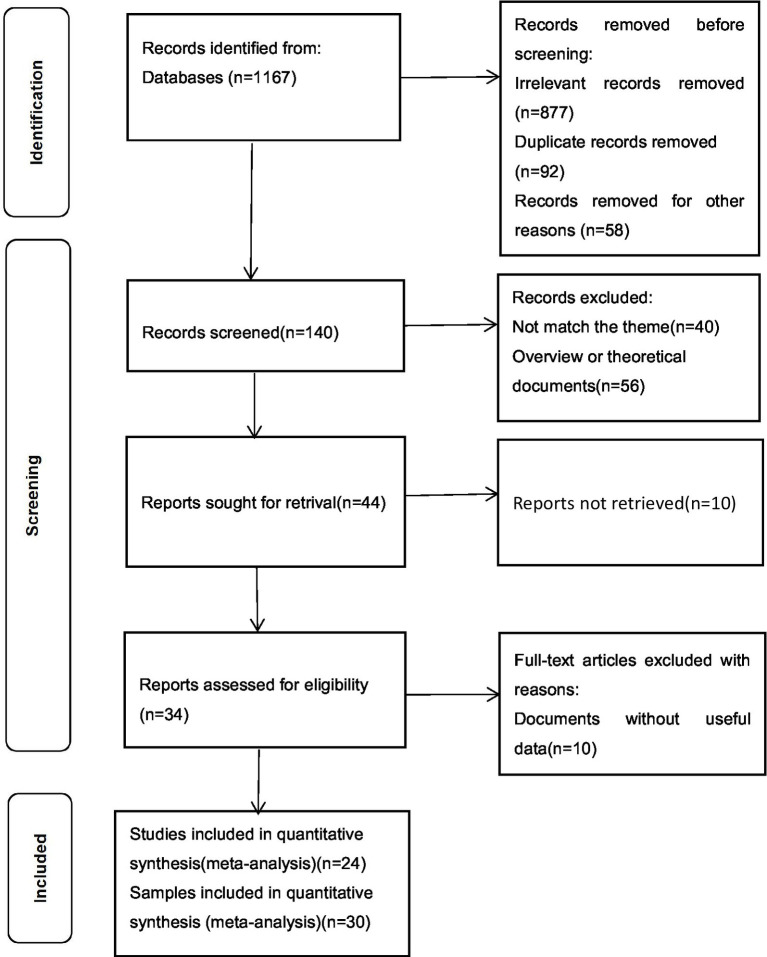
The PRISMA flow chart used to identify studies for detailed analysis of IGD and AG.

The study inclusion criteria were: (i) Studies that simultaneously used IGD and AG scales, and the Pearson product–moment correlation coefficient or the *t*-value and *F*-value that could be converted into *r* were reported; (ii) Studies that reported on sample sizes; (iii) Studies that involved participants who were normal, excluding other groups such as patients and criminals; and iv. For data that were repeatedly published, only one set published in a professional academic journal was chosen. Finally, 24 papers with 30 samples met the meta-analysis criteria. The PRISMA flow chart of the systematic search is shown in Figure 1.

### 2.2. Coding variables

The collected literature were coded by characters, including author names, survey years, publication dates, regional distribution, document types, age of participants, sample sizes, correlation coefficients, measuring tools for IGD and AG as well as the percentage of female students in the overall population (Table 1). Effect values were extracted according to the following criteria: First, the correlation coefficients between IGD and AG were included in the coding. Second, independent samples were coded once. If multiple independent samples were reported at the same time, they were separately coded. Lastly, when calculating the effect value for each category, each original datum appeared only once under each category to ensure the independence of effect value calculation.

**Table 1:** Characteristics of the 30 samples included in the meta-analysis.

Number	Name (year)	Survey year	Journal	Region	Grade^a^	*N*	Female^b^	IGD scale	AG scale	*r*
1	Agarwal et al. (2019) (29)	2017	General	Asia	1	100	Nope	GAS	BPAQ	−0.025
2	Bao (2009) (57)	2007	Dissertation	Asia	1	339	0.379	IADS	BPAQ	0.250
3	Cancer et al. (2021) (18)	2020	General	Asia	2	856	Nope	GAS	BPAQ	0.436
4	Chew et al. (2022) (50)	2020	General	Asia	4	123	0.569	IGDS9-SF	BPAQ	0.290
5	Cui et al. (2006) (39)	2004	General	Asia	2	41	Nope	IADS	BPAQ	0.255
6	Evren1 et al. (2019) (58)	2018	General	Asia	1	987	0.426	IGDS9-SF	BPAQ	0.318
7	Hassan (2021) (51)	2019	General	Asia	1	150	0.500	GAS	BPAQ	0.320
8	Khazaal et al. (2016) (27)	2010	General	Europe	4	3,320	Nope	GAS	Others	0.090
9	Khazaal et al. (2016) (27)	2010	General	Europe	4	2,670	Nope	GAS	Others	0.150
10	Kim et al. (2008) (21)	2006	General	Asia	4	1,471	0.173	Others	BPAQ	0.350
11	Kim et al. (2018) (56)	2016	General	Asia	2	402	0.445	IGDS9-SF	BPAQ	0.320
12	Lemmens et al. (2009) (38)	2007	General	Europe	2	352	0.330	GAS	BPAQ	0.257
13	Lemmens et al. (2009) (38)	2007	General	Europe	2	352	0.330	GAS	BPAQ	0.265
14	Lemmens et al. (2009) (38)	2008	General	Europe	2	369	0.320	GAS	BPAQ	0.205
15	Lemmens et al. (2009) (38)	2008	General	Europe	2	369	0.320	GAS	BPAQ	0.188
16	Mahamid et al. (2020) (54)	2018	General	Asia	4	560	0.693	GAS	BPAQ	0.380
17	Ohno (2021) (52)	2019	General	Asia	4	874	0.486	IGDS9-SF	Others	0.320
18	Su et al. (2018) (59)	2016	General	Asia	2	323	0.529	Others	Others	0.270
19	Teng et al. (2014) (60)	2012	General	Asia	1	211	0.000	Others	BPAQ	0.270
20	Wallenius et al. (2008) (61)	2004	General	Europe	4	478	0.544	Others	Others	0.280
21	Wallenius et al. (2008) (61)	2006	General	Europe	4	316	0.570	Others	Others	0.130
22	Wang (2010) (55)	2008	Dissertation	Asia	1	62	0.403	Others	BPAQ	0.387
23	Wang (2011) (20)	2009	Dissertation	Asia	1	375	0.304	IADS	BPAQ	0.547
24	Wu (2007) (28)	2005	Dissertation	Asia	4	192	0.344	IADS	BPAQ	0.112
25	Yilmaz et al. (2018) (62)	2016	General	Asia	2	276	Nope	Others	BPAQ	0.440
26	Yu et al. (2016) (63)	2014	General	Asia	2	2024	0.494	IGDS9-SF	BPAQ	0.260
27	Yuh (2018) (53)	2016	General	Asia	2	263	0.000	Others	BPAQ	0.260
28	Zhang (2020) (19)	2019	Dissertation	Asia	3	1,080	0.470	Others	Others	0.391
29	Zhang (2020) (19)	2019	Dissertation	Asia	3	1,080	0.470	Others	Others	0.408
30	Zhang (2021) (19)	2019	General	Asia	1	775	0.512	Others	BPAQ	0.610

^a^1 = university students; 2 = middle school students; 3 = primary school students; 4 = mixed. ^b^nope, not reported.

### 2.3. Quality assessment of included studies

Literature quality assessment was performed using the Joanna Briggs Institute (JBI) Critical Appraisal Tool (64). The checklist consists of eight items, each with four options. Questions such as “Were the study subjects and settings described in Detail?” and “Was the statistical analysis appropriate?,” were asked of the studies. The “Yes” option scored 2 points, “Unclear” scored 1 point, while “No” or “Not applicable” scored 0 points. The lowest score was 0 points, while the highest score was 16 points. The literature quality assessment process was independently by two researchers. In case of disagreements, a consensus was reached through discussions. All the 24 studies scored more than 13 points. Quality assessment was not used to exclude any studies but was conducted to enhance the evaluation and discussion.

### 2.4. Effect size calculation

The Pearson product difference correlation coefficient (*r*) was used to calculate the effect sizes (65). The *r* value was transformed by Fisher’s *Z*, calculating weights and 95% confidence intervals based on the sample size Conversion formula: *Zr* = 0.5*ln[(1 + *r*)/(1-*r*)], *VZ* = 1/n-3, *SEz* = sqrt(1/n-3), whereby *Zr* denotes the converted value of the corresponding *r*, *VZ* is the variance, and *SEz* is the standard error.

### 2.5. Data processing and analysis

Data were analyzed using the meta-analysis software, CMA 3.0. To test whether each study result was representative of an estimated sample of the overall effect size, a homogeneity test was performed. The homogeneity test provides a basis for whether the results were fixed-effect or random-effects models. In case of homogenous effect values, the fixed-effect model was selected. If heterogeneity was considerable, a random-effects model was used. The homogeneity test also provides a basis for analysis of moderating effects, while a large heterogeneity indicates the existence of moderating effects (66).

## 3. Results

### 3.1. Effect sizes and the homogeneity test

A total of 24 papers which reported on the relationship between IGD and AG, with 30 sample sizes were identified. A total of 20,790 participants were included in the studies, with the number of subjects ranging from 41 to 3,320 subjects. Table 2 shows 30 independent samples of IGD and AG. The homogeneity test revealed a *Q* statistics value of 483.906, *p* < 0.001, *I*^2^ = 94.007, indicating heterogeneity in the included studies. This may be due to the different measurement tools used in literature, the source of participants and different sample sizes. That is, there may be a moderating effect. Based on the methodology provided by Lipsey and Wilson, the included literature was highly heterogeneous and was analyzed by random models (66).

**Table 2:** Model of the correlation between IGD and AG.

*k*	N	Mean *r* Effect size	95% CI for *r*	Homogeneity test			Tau-squared			Test of null (two-tailed)
				*Q*(r)	*p*	I-squared	Tau-squared	SE	Tau	*Z*-value
30	20,790	0.300	[0.246, 0.353]	483.906	0.000	94.007	0.024	0.009	0.154	10.287***

*p < 0.05, **p < 0.01, ***p < 0.001, the same as follows.

The random model revealed that the correlation between IGD and AG was significant, with correlation coefficients of 0.300, 95%CI [0.246, 0.353]. This relationship is potentially moderate (66). The *Z*-value of IGD and AG relationship was 10.287, *p* < 0.001, indicating that the relationship between IGD and AG is stable (Table 2).

### 3.2. Moderator analysis

As described in section 2.4, the random effects model was applied in the intermediary effects analysis. Meta-ANOVA analysis is suitable for analyzing the moderating effects of categorical variables, such as types of measurement tools, subject groups, and regional differences. In contrast, meta-regression analysis is suitable for analyzing continuous variables’ moderating effects, such as proportions of females and survey year.

### 3.3. Meta-ANOVA analysis

To determine the moderating effects of the relationship between IGD and AG, Meta-ANOVA analysis was performed for the categorical variables (Table 3).

**Table 3:** Region and age and measures moderators of the association between IGD and AG.

	Between-group effect (*Q*_BET_)	*k*		Mean *r* effect size	SE	95% CI for *r*		Homogeneity test within each group (*Q*_W_)
						LL	UL	
Region	16.724***							
Asia		22		0.342	0.007	0.291	0.392	194.324***
Europe		8		0.189	0.004	0.136	0.241	32.349***
Age	19.138***							
University student		8		0.354	0.034	0.207	0.485	118.873***
Middle school student		11		0.292	0.005	0.236	0.347	43.423***
Primary school student		2		0.400	0.001	0.363	0.434	0.220
Mixed		9		0.237	0.011	0.152	0.318	131.651***
Measures of IGD	3.953							
GAS		10		0.235	0.014	0.147	0.319	138.872***
IADS		4		0.307	0.059	0.064	0.515	39.563***
IGDS9-SF		5		0.293	0.001	0.264	0.323	4.445
Others		11		0.354	0.012	0.272	0.430	119.511***
Measures of AG	1.068							
BPAQ		22		0.317	0.009	0.258	0.374	210.260***
Others		8		0.258	0.015	0.159	0.352	173.801***

***p < 0.001.

The homogeneity test (*Q* = 16.724, *df* = 1, *p* < 0.001) revealed that regions moderated this correlation. Correlation coefficients between IGD and AG for Asian and European subjects were 0.342 (95% CI = [0.291,0.392]) and 0.189 (95% CI = [0.136,0.241]), respectively, indicating that *r*_Europe_ < *r*_Asia_.

The homogeneity test (*Q* = 19.138, *df* = 3, *p*<0.001) showed that age had moderating effects on this correlation. The correlation coefficients between IGD and AG for university, middle school and primary school students were 0.354 (95% CI = [0.207, 0.485]), 0.292(95% CI = [0.236, 0.347]) and 0.400 (95% CI = [0.363, 0.434]), respectively, indicating that *r*_middle school students_ < *r*_university students_ < *r*_primary school students_.

The homogeneity test (*Q* = 3.953, *df* = 3, *p* > 0.05) showed that measurement tools for IGD did not have moderating effects on this correlation, and the relationship between IGD and AG was not affected by measurement tools for IGD.

The homogeneity test (*Q* = 1.068, *df* = 1, *p* > 0.05) demonstrated that measurement tools for AG had no moderating effects on this correlation, and the relationship between IGD and AG was not affected by measurement tools for AG.

### 3.4. Meta-regression analysis

Meta-regression analysis was performed to determine whether the continuous variables had significant effects on the relationship between IGD and AG. Results showed that: (i) The effect of sex on the relationship between IGD and AG was not significant. It showed the proportion of women did not significantly influence the relationship between IGD and AG (*Q*_Model[1, k = 24]_ = 0.490, *p* > 0.05; Table 4). (ii) The year was a significant factor moderating the relationship between IGD and AG. Meta-regression analysis showed (Table 4) that year significantly affected the relationship between IGD and AG (*Q*_Model[1, k = 30]_ = 5.380, *p* < 0.05). Specifically, as the years increases, the correlation coefficient between IGD and AG also increases.

**Table 4:** Meta-regression analysis of gender and survey year.

Variables	Parameter	Estimate	SE	*Z*-value	95%CI for b	
					LL	UL
Female (%)	*β_0_*	0.121	0.173	0.700	–0.219	0.461
	*β_1_*	0.278	0.075	3.690	0.130	0.425
	*Q_Model (1, k = 24)_* = 0*.490, p ﹥ 0.05*					
Survey year	*β_0_*	0.011	0.005	2.320	0.002	0.021
	*β_1_*	−22.159	9.684	−2.290	−41.139	−3.179
	*Q_Model (1, k = 30)_* = *5.380, p < 0.05*					

### 3.5. Assessment of publication bias

**Figure 2:**
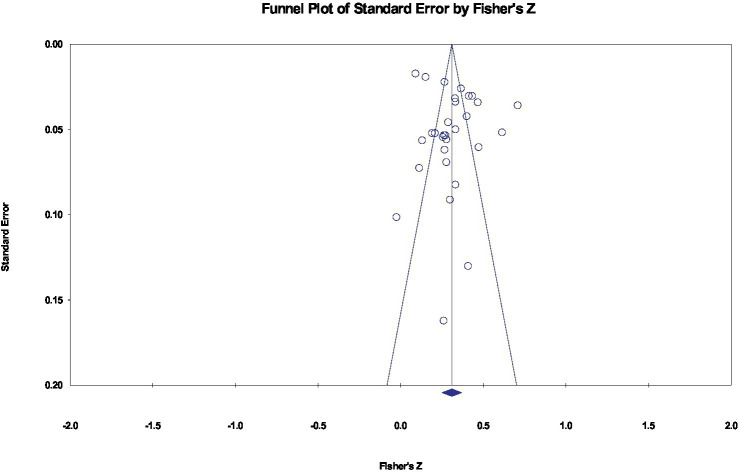
Funnel plot of effect sizes of the correlation between IGD and AG.

To determine whether the results were biased due to effect sizes from various sources, a funnel plot was drawn (Figure 2). The 30 effect sizes were symmetrically distributed on both sides of the average effect size, and Egger’s regression test (67) did not reveal a significant bias [*t*_(28)_ = 1.387, *p* = 0.176 > 0.05]. To test for publication bias, this study calculated that the *Z* = 38.953 (*p* < 0.001) of Classic Fail-safe N. When 1820 missed studies were included, the analysis result was no longer significant (68). These findings showed that the overall correlation between IGD and AG was stable.

## 4. Discussion

### 4.1. The positive relationship between IGD and AG

Our results revealed a significant correlation between IGD and AG, consistent with previous studies (21, 54, 56). Yuh reported that individuals’ AG can predict their IGD (53). From the Social Information Processing (SIP) Model of AG, people who perpetrate aggressive behaviors tend to choose negative social cues and hold attributional biases when facing ambiguous adverse events in life (44). Most of them may lack social problem-solving skills (69), have a low self-esteem (70) and experience negative interpersonal relationships (71). To escape the unsatisfactory real life, they are more likely to dedicate themselves to virtual Internet games, which may make them feel powerful to overcome difficulties and get rid of loneliness (72). From another perspective, most online games contain fighting, competition and violence, which promote the gamers’ violent tendencies (73). The social learning theory suggests that people’s aggressive behaviors are learned from observation (74). People are not born to be aggressive, but learn to behave violently from external circumstances. For instance, individuals who grew up in parenting-conflict environments might exhibit more aggressive behaviors than others (75, 76). From the perspective of cognitive-contextual framework, people also learn how to use force to solve difficulties in the violent gaming world. They may consider AG as the best solution to a problem or to acquire what they want (77). Besides, in Internet games, people can achieve extremely positive emotional experiences through violence without being actually punished, which may cause them underestimate the AG consequences and reinforce the urge to commit AG in real life (78). Therefore, people who are addicted to Internet games may show more AG in daily life.

### 4.2. Moderating effects

We found that region moderated the relationship between IGD and AG. Specifically, the correlation coefficient between IGD and AG in Asian participants was significantly higher compared with that of European participants. Compared with the European individualistic cultures, individuals in Asian collectivist cultures are less self-contained (79). To regulate their behaviors, they are more likely to be influenced by the context and surrounding people (80). Therefore, in the face of violent contexts in Internet games, young people in Asia may have a more challenging time maintaining their self-awareness and instead imitate those behaviors. Besides, studies have shown that individuals are weaker in self-monitoring in collectivist cultures than in individualistic cultures (81). Collectivist cultures in the Asian region encourage harmony and alignment to others. Individualized emotions, such as anger, are often suppressed. Compared with Western regions that value individuality and have fewer inhibitions on people’s expression, individuals with IGD in Asia may find it harder to control themselves, thereby expressing repressed negative emotions and exhibiting aggressive behaviors. Compared to Asian countries, Western countries were the first to study and treat IGD. They established a better supervision mechanism, such as the Entertainment Software Rating Board (ESRB), which set a game rating system to help parents prevent and control minors’ exposure to unhealthy games. Thus, teenagers who play games in Western countries may be less likely to be exposed to and learn about violent and aggressive behaviors in games. Finally, the moderating effects may be influenced by sample distribution. A total of 30 samples were included in this study (8 in Europe and 22 in Asia). The imbalance in sample distribution may affect the relationship between IGD and AG.

In addition, the results revealed that age moderated the relationship between IGD and AG. The correlation coefficient between IGD and AG was highest among primary school students, followed by college students, and finally middle school students. People who are forced to stop Internet gaming may exhibit anger and aggression (82). Unfortunately, compared with college and middle school students, primary school students have the weakest self-control abilities (83), which makes them exhibit severe aggressive behaviors negative emotions and play games as a means to escape from these challenges. Thus, the vicious circle is more likely to occur among primary school students. We also found that college students perform worse than middle school students regarding the relationship between IGD and AG. That may be because when young people enter colleges, their parents reduce rule setting and discipline (84), making them relay on self-monitoring and self-management. Therefore, it may be challenging for young adults to prevent themselves from engaging in Internet games and problematic behaviors. In addition, our sample sizes for different age groups were not even. For instance, the sample size for primary school students was small, which may have affected the moderating effects of age on the relationship.

For the moderating role of survey year, results showed that the relationship between IGD and AG increased over time, which is consistent with findings from previous studies that IGD can predict aggressive behaviors, and that the relationship between the two is longitudinal and synchronous (60, 85). In the past two decades, advances in network technologies have been significant. The Internet has increasingly become the most commonly used work and entertainment tool for the public. According to the Digital 2022: Global Overview Report data, between 2012 and 2022, the number of Internet users worldwide increased from 2.18 billion to 4.95 billion (86). During the period from 1998 to 2016, the prevalence of IGD increased from 0.7 to 15.6% (87). Spending too much time on the screen for children and adolescents may promote their psychological and behavioral problems (88). Therefore, with development of the Internet, young people’s addiction to Internet games may increase the correlation between IGD and AG. In addition, teenagers and young adults experience more stress and anxiety compared to previous years, which may emotional and behavioral distress (89). The increasing stress levels may be a potential reason for strengthening the relationship between IGD and AG.

### 4.3. Limitations and future studies

This study applied the Egger’s publication bias test which revealed that there was no significant publication bias in the included studies and that the meta-analysis results were stable. Thus, compared with results from single sample groups, the present results are more reliable, representative, and authentic.

However, there are still some limitations to this meta-analysis. First, we only included non-clinical samples of AG and IGD. Studies investigating IGD among individuals diagnosed with behavioral disorders such as AG disorders are few. We believe that serials of data are also crucial for the theoretical and practical areas. Therefore, more research is needed to explore the causal relationships between the two *via* experimental design and use clinical samples to test the relationships between IGD and AG. In addition, this study only examined limited moderating variables, such as age and region. Many potential factors that may influence the relationships between IGD and AG should be explored further.

From the perspective of implementing interventions, we found differences in IGD and AG among age subgroups. Compared with primary and secondary school students, college students have more time for Internet games. Moreover, compared with measures such as real-name authentication for minors to prevent IGD, the intervention measures for IGD among college students are not effective. Policymakers should pay attention to the problem of IGD among primary and secondary school students as well as college students. In addition, schools should develop supportive programs to assist individuals who are addicted to IGD and AG. Since individuals who are addicted to Internet games are seeking self-efficacy on the Internet, schools can improve multiple assessment systems for students so that they can find self-worth in real school-life and provide professional services, such as psychological counseling and group counseling activities for individuals with AG to alleviate students’ symptoms of IGD and AG.

## 5. Conclusion

In this study, we found a moderate positive correlation between IGD and AG. That is, people with a higher level of IGD may show more AG. In addition, people with more aggression behaviors may have higher level of IGD. Furthermore, the relationship between IGD and AG is moderated by several variables including region, age, and survey year. The association between IGD and AG is stronger in Asia than in Europe. The correlation coefficient between IGD and AG is in the order of primary school, college, and middle school students. Finally, the relationship between IGD and AG increases with the increase in survey year.

## Data availability statement

The original data presented in the study is included within the article. Further inquiries can be directed to the corresponding author.

## Ethics statement

Ethical review and approval was not required for the study on human participants in accordance with the local legislation and institutional requirements. Written informed consent from the participants was not required to participate in this study in accordance with the national legislation and the institutional requirements.

## Author contributions

SL and YZ designed and supervised the study, and did all statistical analyses. ZW did the literature search and drafted the first version of the article. MX, XW and XM contributed to review and revision. All authors contributed to the article and approved the submitted version.

## Funding

This research was sponsored by the Project of Social Science Foundation of Xinjiang Uygur Autonomous Region (22CMZ018) and the Project of Center for Teacher Education Research in Xinjiang (ZK202232B) and the project of Doctoral Research Startup Fund at Xinjiang Normal University (XJNUBS201908).

## Conflict of interest

The authors declare that the research was conducted in the absence of any commercial or financial relationships that could be construed as a potential conflict of interest.

## Publisher’s note

All claims expressed in this article are solely those of the authors and do not necessarily represent those of their affiliated organizations, or those of the publisher, the editors and the reviewers. Any product that may be evaluated in this article, or claim that may be made by its manufacturer, is not guaranteed or endorsed by the publisher.
